# Analysis of expression of vitamin E-binding proteins in H_2_O_2_ induced SK-N-SH neuronal cells supplemented with α-tocopherol and tocotrienol-rich fraction

**DOI:** 10.1371/journal.pone.0241112

**Published:** 2020-11-24

**Authors:** Aishatu Ali Chiroma, Huzwah Khaza’ai, Roslida Abd. Hamid, Sui Kiat Chang, Zainul Amiruddin Zakaria, Zaida Zainal

**Affiliations:** 1 Department of Biomedical Sciences, Faculty of Medicine and Health Sciences, Universiti Putra Malaysia, Selangor, Malaysia; 2 Key Laboratory of Plant Resources Conservation and Sustainable Utilization, Key Laboratory of Post-Harvest Handling of Fruits, Ministry of Agriculture, South China Botanical Garden, Chinese Academy of Sciences, Guangzhou, China; 3 Integrative Pharmacogenomics Institute (IPROMISE), Faculty of Pharmacy, Universiti Teknologi MARA, Selangor, Malaysia; 4 Nutrition Unit, Product Development and Advisory Services Division, Malaysian Palm Oil Board, Selangor, Malaysia; Xiangtan University, CHINA

## Abstract

Natural α-tocopherol (α-TCP), but not tocotrienol, is preferentially retained in the human body. α-Tocopherol transfer protein (α-TTP) is responsible for binding α-TCP for cellular uptake and has high affinity and specificity for α-TCP but not α-tocotrienol. The purpose of this study was to examine the modification of α-TTP together with other related vitamin E-binding genes (i.e., *TTPA*, *SEC14L2*, and *PI-TPNA*) in regulating vitamin E uptake in neuronal cells at rest and under oxidative stress. Oxidative stress was induced with H_2_O_2_ for an hour which was followed by supplementation with different ratios of α-TCP and tocotrienol-rich fraction (TRF) for four hours. The cellular levels of vitamin E were quantified to determine bioavailability at cellular levels. The expression levels of *TTPA*, *SEC14L2*, and *PI-TPNA* genes in 0% α-TCP were found to be positively correlated with the levels of vitamin E in resting neuronal cells. In addition, the regulation of all the above-mentioned genes affect the distribution of vitamin E in the neuronal cells. It was observed that, increased levels of α-TCP secretion occur under oxidative stress. Thus, our results showed that in conclusion vitamin E-binding proteins may be modified in the absence of α-TCP to produce tocotrienols (TCT), as a source of vitamin E. The current study suggests that the expression levels of vitamin E transport proteins may influence the cellular concentrations of vitamin E levels in the neuronal cells.

## Introduction

Vitamin E is divided into two iso-forms: tocopherols and tocotrienols. They consist of eight different isomers: the α, β, γ, and δ-tocopherols and the α, β, γ, and δ-tocotrienols. All eight forms are antioxidants, able to scavenge and quench free radicals by donating an electron to neutralize reactive free radicals. Of all the naturally occurring and synthetic vitamin E family members, only α-tocopherol is preferentially retained in the human plasma, and therefore, is considered as a major fat-soluble antioxidant present in higher concentrations in mammalian tissues [1]. However, tocotrienols have shown to be better than tocopherols in scavenging peroxyl radicals due to a more even distribution of tocotrienols in the phospholipid bilayer. Hence, tocotrienols interact more effectively with lipid peroxyl radicals in membrane environments. In addition, to their antioxidant activities, studies have demonstrated that vitamin E possesses various important functions, such as in enhancing immune function, cell signaling, regulation of gene expression, cell homeostasis, and other metabolic processes [2–4]. Vitamin E plays an important role at the level of plasma membranes; modulating signaling pathways and gene regulation [5]. Vitamin E is also a powerful mediator and functions in protecting the nervous system against oxidative stresses due to free-radical attack. Vitamin E has an important role in preventing neurodegenerative diseases, such as Alzheimer’s disease, via its supplementation [6] as well. Tocopherols and tocotrienols protect neuronal cells against cell death initiated by oxidative stress, and tocotrienols have demonstrated enhanced protection against glutamate-induced injury in neuronal and astrocyte cells [7–9].

By virtue of its lipophilic character, tocopherol requires a protein to facilitate its transport through the fluid milieu of the cytosol. The presence of α-tocopherol transfer protein (α-TTP) in the liver preferentially binds tocopherol with high affinity, which catalyzes its transport to the plasma membrane [10, 11]. α-TTP and ATP-binding cassette transporter A1 (ABCA1) then incorporate α-tocopherol into nascent lipoproteins, transporting it to other tissues through blood circulation [12]. α-TTP has been shown to be expressed in various tissues such as the liver, cerebellum, and pre-frontal cortex of the brain, kidneys, lung [13], placental trophoblasts, and in murine uterus [14–16], with the liver having the greatest expression. Even though, α-TTP is a key regulator of α-tocopherol (α-TCP), the mechanism that regulates the tissue-specific expression of this protein is not completely understood. Studies *in vivo* have demonstrated that α-TCP status may regulate the expression of α-TTP where there is deficiency of α-TCP and resulted in low levels of α-TTP [17, 18] production. It has been shown that vitamin E repletion reduced α-TTP levels while depletion did not result in an increase in the level of α-TTP mRNA levels. Several *in vivo* [19–21] and *in vitro* [22, 23] studies have shown that oxidative stress up-regulated the α-TTP levels. On the other hand, *in vivo* studies have also reported that oxidative stress downregulated α-TTP levels [19, 24].

α-TTP selectively binds α-TCP for cellular uptake, where it has strong specificity and high affinity for α-TCP and poorly binds α-tocotrienol (α-TCT) [25, 26]. Palm oil, which is the main source of vitamin E among the Malaysians, contains high level of tocotrienols; the distribution of vitamin E in palm oil is 30% tocopherols and 70% tocotrienols [27]. Therefore, the study on the mechanisms of α-TTP in maintaining vitamin E homeostasis in plasma has become the main interest of the present study. The importance of α-tocopherol homeostasis became apparent when it was observed that mutations in the α-TTP gene led to ataxia with vitamin E deficiency (AVED)—an autosomal recessive disease in which the degeneration of neurons results in progressive spino-cerebellar ataxia [28] and retinitis pigmentosa [29]. Efforts to understand the uptake of non-tocopherol vitamin E is important since, it was established that patients with Alzheimer’s disease (AD) is deficient of vitamin E [23]. Hence, the presence of other tocopherol-associated proteins (TAP) and tocopherol-binding proteins (TBP) to achieve optimum uptake of vitamin E in neuronal cells is beneficial in the treatment of AD.

This study hypothesized that under severe α-TCP deficiency, the transport proteins may be modulated. Understanding the concerted effort between proteins involved in vitamin E uptake in the neuronal cells warrants further investigation. Therefore, in addition to α-TTP; two other proteins having strong relationships with vitamin E uptake: phosphatidyl-inositol transfer protein (PI-TP) and supernatant protein factor (SPF). Both of which are involved in vitamin E homeostasis–has also became of interest to this study in order to have new insights about the uptake functions of these proteins towards other vitamin E isomers [11, 30].

The main aim of the present study was to determine the expression of TTPA, SEC14L2, and PI-TPNA genes for α-TTP, PI-TP, and SPF proteins, respectively, in regulating vitamin E uptake in neuronal cells during resting and under oxidative stress upon supplementation with different ratios of the α-TCP and tocotrienol-rich fraction (TRF) of palm oil. This study also aimed to explain the mechanisms of absorption of tocotrienol under oxidative stress as well as α-TCP deficiency. Furthermore, α-TTP and other vitamin E-associated proteins, namely PI-TP and SPF, are transport proteins have strong association with vitamin E uptake.

## Materials and methods

### Chemicals and reagents

The palm oil TRF and α-TCP used in this study were supplied by Sime Darby Bioganic Private Limited, Kuala Langat, Selangor, Malaysia. Culture flasks, 96-well plates, and pipettes were obtained from TPP (Trasadingen, Switzerland). Minimum essential medium (MEM) culture media, penicillin/streptomycin, trypsin, 4-(2-hydroxyethyl)-1-piperazineethanesulfonic acid (HEPES) buffer, and phosphate-buffered saline (PBS) were purchased from Gibco (Invitrogen, USA). 3-(4,5-Dimethylthiazolyl-2)2,5-diphenyltetrazolium bromide (MTT) powder was obtained from PhytoTechnology Laboratories (Flint St, Kansas, USA). Dimethyl sulfoxide (DMSO) was purchased from Sigma Aldrich (St. Louis, MO, USA). RNA extraction kit was purchased from Favorgen (USA), comprising FARB buffers and qPCRBIO cDNA synthesis kit, and qPCRBIO SyGreen Mix Lo ROX were purchased from PCR Biosystems (UK). Reverse and forward primers and housekeeping genes were purchased from GeneCopoeia (USA). All other chemicals used in this study were of analytical grade.

### Cell culture

The neuroblastoma cell line, SK-N-SH (ATCC^®^ HTB-11^TM^), was purchased from the American Type Culture Collection (ATCC) (Manassas, VA, USA). SK-N-SH is a neuroblastoma cell line that displays epithelial morphology and grows in adherent culture. This neuronal cell line was used to model an insulin resistance condition since it expresses the components of the insulin signaling pathway [31, 32]. The cells were maintained in minimum essential medium (MEM) culture media added with 10% fetal bovine serum (FBS), endothelial cell growth supplement, and 1% penicillin/streptomycin, respectively, in a humidified atmosphere of 95% air and 5% CO_2_ at a temperature of 37°C. In all experiments, confluent neuronal cells corresponding to passages 5–8 were used. The growth medium (MEM) was prepared according to the procedures recommended by the American Tissue Cell Culture.

### Induction of oxidative stress and time course study

First, the neuronal cells were cultured in a multi-well plate and incubated for 24 hours. Prior to inducing oxidative stress using hydrogen peroxide, the old media was removed and 180 μL of complete MEM was added. That was followed by exposure of the cells to different concentrations of H_2_O_2_ (25, 50, 100, 150, and 200 μM) for an hour. The experiment was carried out in three replicates. The H_2_O_2_ solution was diluted in growth media to the designated concentration, with the only 1 μL of H_2_O_2_ solution at different concentrations added to each well to achieve the correct final concentration. Control samples received the corresponding volume of media.

Neuronal cell viability was assessed by 3-(4,5-dimethylthiazolyl-2)2,5-diphenyltetrazolium bromide (MTT) assay according to Musa et al. [8], with slight modifications. After 24 hours, 100 μL of MTT (5 mg/mL) was added to each well and incubated with the cells for 4 hours at 37°C in a 5% CO_2_ atmosphere. Subsequently, 200 μL of DMSO was added to each well to dissolve the formazan crystals. The cells were then incubated for another 5 minutes in the dark at room temperature, mixed thoroughly, and then 100 μL from each well was transferred to a 96-well plate. The absorbance value was measured using an enzyme-linked immunosorbent assay (ELISA) reader (BIO-TEK EL×800, USA) at 570/630 nm. A graph of cell viability (%) against H_2_O_2_ concentration (μM) was plotted to determine the IC_20_. This experiment was repeated three times to observe the consistency and reproducibility of the results. Once the IC_20_ was determined, that concentration was used for the time course study. For the time course study, the neuronal cells were treated with the IC_20_ concentrations of H_2_O_2_ and incubated for 0, 1, 2, and 3 h, respectively. The duration at which the IC_20_ was achieved was then used for all subsequent experiments. The IC_20_ was needed in this study in order to ensure minimal cell injury when the cells were not at rest, and it was expected to lead to the expression of genes that facilitate the uptake of vitamin E more readily compared to when the cells were at rest.

### Preparation of vitamin E

Palm oil TRF contents (25% α-tocopherol and 75% tocotrienols) and purity were confirmed according to our previous studies [8, 9]. α-TCP was prepared in 100% ethanol at a concentration of 200 ng/mL. Our previous studies demonstrated that 200 ng/mL of α-TCP and TRF was the optimum concentration to produce an effective treatment for the cells to recover from glutamate insult [8, 9]. The vitamin E was stored at 4°C until further analyses.

### Gene expression analysis using real time PCR

#### Treatment of neuronal cells with hydrogen peroxide and vitamin E

Before H_2_O_2_ treatment, neuronal cells were seeded in a T_25_ flask for 24 hours to achieve confluency. Then, the old media was removed, and the cells were washed three times with 1 mL of phosphate buffered saline (PBS). Then, the PBS was removed, and 4 ml of new media was incorporated to each flask. Subsequently, 15 μL of H_2_O_2_ at a concentration of 25 or 50 μM was added into each flask. The flask was incubated for an hour at 37°C with 5% CO_2_ and 95% air. After having incubated for an hour, the cells were ready for vitamin E treatment. Different volumes of α-TCP and TRF at a concentration of 200 ng/mL with different ratios (Table 1) were added into each flask. The flasks were incubated for four hours. Appropriate solvent was used as vehicle control.

**Table 1:** Experimental design of different percentages/ratios of α-TCP and TRF treatment.

Percentages Ratios
(100% α-TCP to 0% TRF) = (4 α-TCP:0 TRF)
(75% α-TCP to 25% TRF] = (3 α-TCP:1 TRF]
(50% α-TCP to 50% TRF) = (2 α-TCP:2 TRF)
(25% α-TCP to 75% TRF) = (1 α-TCP:3 TRF)
(0% α-TCP to 100% TRF) = (0 α-TCP:4 TRF)

#### Total RNA isolation and real-time PCR

Total RNA was extracted using the commercial FavorPrep^TM^ Blood/Cultured Cell Total RNA Mini kit (Favorgen, UK) according to the manufacturer’s instructions. The isolated RNA was then reverse transcribed to cDNA by employing real-time polymerase chain reaction (RT-PCR). cDNA was synthesized using a PCR Biosystems cDNA synthesis kit (PCR Biosystems, UK). Quantitative RT-PCR amplification was carried out with MiniOpticon™ real-time detection system, Bio-Rad (MJ Research, Waltham, MA, USA) using qPCRBIO SyGreen, forward primer (10 μM) and reverse primer (10 μM) specific for each gene of interest, PCR-grade distilled water nuclease-free water and, lastly, template cDNA (100 ng/μL) according to the manufacturer’s instructions. The sequences of the oligonucleotide primers for supernatant protein factor *(SEC14L2)*, phosphatidylinositol transfer protein *(PI-TPNA)*, α-tocopherol transfer protein *(TTPA)*, and (*GAPDH)* are shown in Table 2. PCR conditions for these primers were 95°C for one min, 95°C for 5 second and 60°C for 20 second over 40 cycles.

**Table 2:** Specific primers for *TTPA*, *PT-TPNA*, *SEC14L2*, and *GAPDH*.

Genes	Forward and Reverse Primers
*TTPA*	5′ TGT CTG GGA AAT GCT GAA GC 3′
	5′ AGT CCT CAG ATC CAG GGA TC 3′
*PI-TPNA*	5′ GCT GCT CAA GGA GTA TCG AGT 3′
	5′ GGG TAC TTT GCT CTG CAG GT 3′
*SEC14L2*	5′ TGG AGC GGA TGT TGG TTT 3′
	5′ TTG GCA TGA ATG AAG CTG TAG G 3′
*GAPDH*	5′ CAACTACATGGTTTACATGTTC 3′
	Reverse = 3′ GCCAGTGGACTCCACGAC 5′

In each independent experiment, target and reference gene cDNA were derived from similar extracted RNA and run simultaneously in the RT-PCR. Three independent experiments were carried out and the collected quantitative data were averaged based on quantification cycle (C_q_) values, which were used to calculate the fold expression ratio. *GAPDH* was included as the reference gene for normalization of the target genes and for compensation of inter-PCR variation between each RT-PCR experiment.

### Extraction of cellular vitamin E

Cellular α-TCP and TRF contents (representative trace) were determined using high-performance liquid chromatography (HPLC). Confluent neuronal cells were incubated for 16 hours with their respective media, after which α-TCP and TRF were extracted. First, the media was discarded, and the cells were washed twice with 1 ml PBS. Then, trypsin (0.25%) was added to the cells, and incubated for five minutes. After that, 2 ml of media was added into the wells, and the cell suspension was transferred into a centrifuge tube and centrifuged at 1000 rpm for six minutes. The supernatant was discarded, and 1 ml of PBS was added into the tube. The cells were again centrifuged at 1000 rpm for six minutes to obtain pure pellets. For vitamin E extraction, the supernatant was removed, and 1 ml of distilled water, 1 ml ethanol, and 2 ml hexane (1:1:2) were added to the centrifuge tube. The tube was vortexed until two layers were apparent. Further separation was then carried out by centrifuging at 1000 rpm for six minutes. Finally, the upper layer was collected and dried under N_2_. The extracted vitamin E was stored at 4°C until HPLC analysis.

### HPLC analysis of vitamin E content

Chromatographic separation of α-TCP and tocotrienols was carried out using Agilent HPLC 1200 system (Agilent Technologies, Waldbronn, Germany) with a quaternary pump solvent delivery system, degasser and autosampler at 35°C using a YMC C18 silica gel column (ZORBAX Rx-SIL, 4.6 mm × 250 mm; Agilent Technologies, Palo Alto, CA, USA). The mobile phases used were 99.5% hexane and 0.5% iso-propanol at a flow rate of 1.0 ml/minute. The injection volume was 10 μL. The detection was set at 295 nm excitation and 325 nm emission. Total vitamin E content was quantified using the calibration curves of corresponding standard solution containing α-TCP, α-tocotrienol, β-tocotrienol, γ-tocotrienol and δ-tocotrienol.

### Statistical analysis

All data were analyzed using the Statistical Package for the Social Sciences (SPSS) version 22 (SPSS Inc., Chicago, IL, USA). Each treatment in a 96-well plate was carried out using at least three independent replicates. Data were expressed as mean ± SD. One-way analysis of variance (ANOVA) followed by Bonferroni’s post hoc test was used to compare the experimental means. *p* < 0.05 was considered statistically significant.

## Results and discussions

### Dose-response and time course study

**Fig 1:**
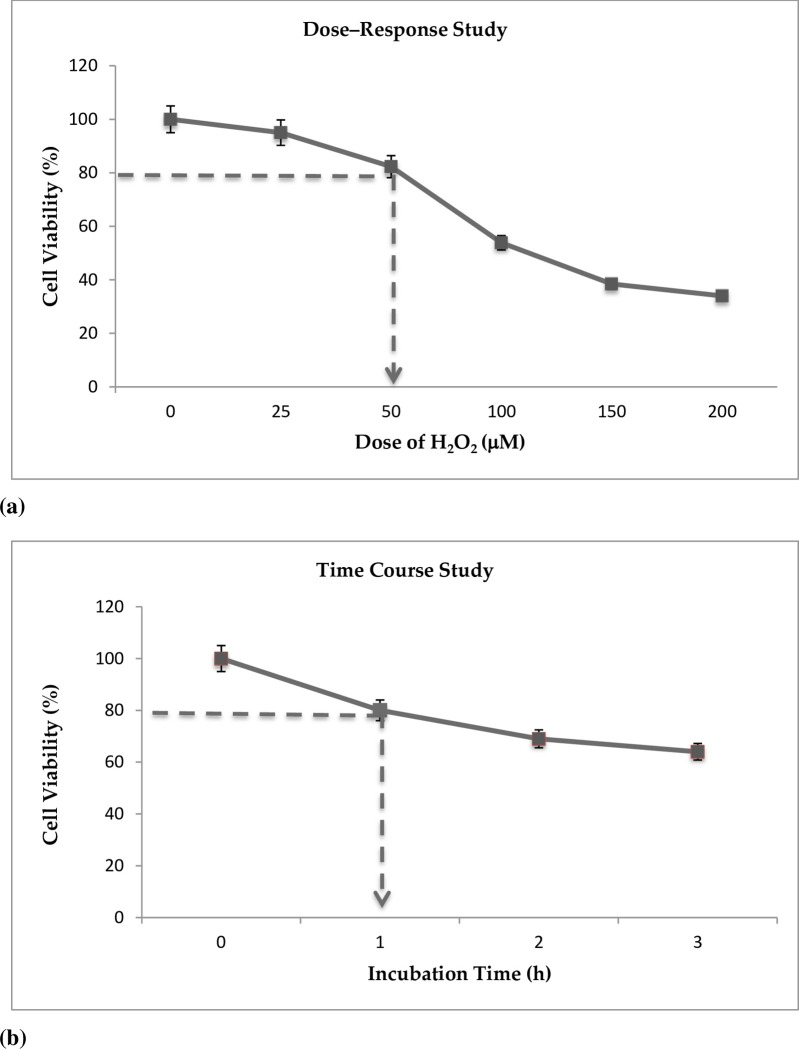
(**a**) Determination of neuronal cell viability following H_2_O_2_ exposure using an MTT assay. Results are expressed as % of control viability (100%) and are shown as mean ± SEM (*n* = 3 experiments performed in triplicate). (**b**) Graph of incubation time against cell viability. Results are expressed as % of control viability (100%) and shown as mean ± SEM (independent experiments performed in triplicate).

Hydrogen peroxide (H_2_O_2_) serves as an intracellular messenger and acts as a cell signaling molecule under normal conditions. However, H_2_O_2-_in excess caused toxicity in the neuronal cells and eventually led to oxidative stress. *In vitro* toxicity is a well-established model for generating oxidative stress. A dose-response study was carried out in order to determine the tolerance level of neuronal cells against H_2_O_2_ challenge. In this experiment, the IC_20_ of H_2_O_2_ toxicity to induce injury in the neuronal cells was determined. Fig 1A demonstrates that the toxicity of H_2_O_2_ was dose dependent; cell survival decreased as the concentration of H_2_O_2_ was increased.

From the experiment, 80% of neuronal cells survived when exposed to 50 μM H_2_O_2_. Therefore, the IC_20_ for neuronal cells was determined as 50 μM H_2_O_2._ and this dosage (50 μM) was used for the subsequent time course study. The time course study was carried out using four incubation periods: 0, one, two, and three hours. This test was conducted to determine the incubation time for neuronal cells against H_2_O_2_ toxicity. Fig 1B shows the incubation time for neuronal cells when challenged with 50 μM H_2_O_2_; it was observed that the time taken for the cells to survive at 80% was one hour. Similar results have previously been reported where it was found that 16–18 hours exposure of cerebellar granule neurons (CGNs) to 50 μM H_2_O_2_ induced approximately 80% cell death [33]. In another study, 50 μM H_2_O_2_ induced 34% cell death, where 66% of the cells were viable in CGNs upon exposure for 20 minutes [34].

Micromolar concentrations of H_2_O_2_ have shown to cause neuronal cell death as a result of the excessive accumulation of reactive oxygen species (ROS), causing an increase in intracellular free Ca^2+^ which, in turn, leads to mitochondrial dysfunction, irreversible organelle and membrane damage and, finally, cell death. Thus, based on the dose-response and time course study, 50 μM of H_2_O_2_ and an hour of incubation was used to induce oxidative stress in neuronal cells throughout this study.

### Gene expression analysis

Vitamin E absorption occurs in the upper and middle parts of the small intestine. The micelles of vitamin E passively diffuse through the brush border of intestinal epithelial cells. They then enter the enterocytes, and the vitamin E is packaged in chylomicrons before being permitted to enter the circulation. Vitamin E is transported in the plasma via lipoproteins—namely: high-density lipoprotein (HDL), low-density lipoprotein (LDL), and very-low-density lipoprotein (VLDL) [1]. It is the major lipid soluble antioxidant in vertebrates while, α-TTP is a 32 kDa soluble protein that plays a key role in vitamin E homeostasis. The transfer of α-TCP into nascent VLDL mediated by α-TTP is the key determining factor(s) of plasma α-TCP concentration in the humans. This was reported from the patients that were suffering from a decreased level of plasma and tissue α-TCP and ataxia with isolated vitamin E deficiency (AVED), when mutations in α-TTP had occurred. Studies have demonstrated that *Ttpa*^*−/−*^ mice showed low vitamin E levels, ataxic phenotype, and had an increase of markers of oxidative stress in the plasma, brain, heart, liver, and uterus [13, 35].

Similar results have been reported in the past by Copp et al. [35] and Hosomi et al. [36], as treated neuronal cells were found to express *α-TTP*, *SPF*, and *PI-TP* genes. α-TTP was found to be expressed in Purkinje cells as well as in pyramidal cells of the hippocampus [35] in Alzheimer’s disease. Hosomi et al. [36] also reported the expression of α-TTP in brain. In the present study, the effects of induced oxidative stress and vitamin E supplementation on the modification/expression of *TTPA*, *SEC14L2*, and *PI-TPNA* genes in neuronal cells were evaluated. The expression of the genes that was induced by oxidative stress were dose dependent with decreasing percentage of α-TCP and increasing percentage of TRF. The highest fold change was mostly observed in 0% α-TCP and where 100% of tocotrienol was available in the cells.

Ulatowski et al. [23] demonstrated that oxidative stress increased *TTPA* expression in astrocyte cells. This suggests that α-TCP is distributed by the *TTPA*-expressing astrocytes in order to protect neurons from oxidative stress-induced damages. Likewise, α-TTP expression was up-regulated during oxidative stress in the human choriocarcinoma cells [22] and in a rat model with type II diabetes [19].

### α-tocopherol transfer protein gene (TTPA) expression in resting and H_2_O_2_-induced neuronal cells

**Fig 2:**
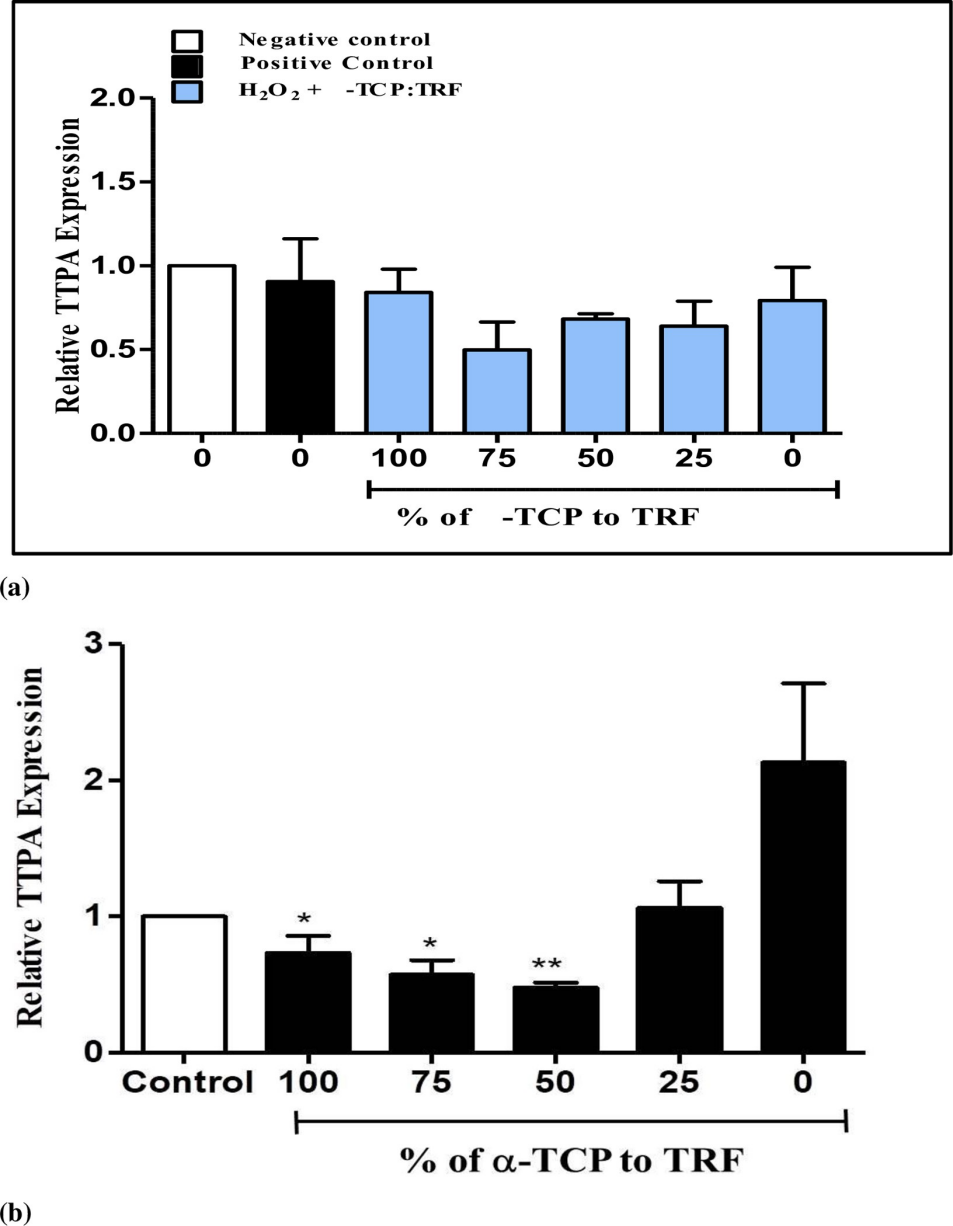
Quantification of *TTPA* gene expression in (**a**) neuronal cells treated with 50 μM H_2_O_2_ and (**b**) neuronal cells rested for an hour followed by supplementation with different percentages 200 ng/mL α-TCP and TRF for 4 hours, after which RNA was isolated. Negative control is untreated cells while positive control is cells treated with H_2_O_2_. **p < 0.01, *p < 0.05 vs. 0% α-TCP; determined by one-way ANOVA with a Tukey’s post hoc test. Fold change of *TTPA* was normalized to *GAPDH*. Results are expressed as mean ± SEM of three independent experiments.

The expression of *TTPA* in H_2_O_2_-induced neuronal cells was supplemented with different percentages of α-TCP and TRF (100% and 0%; 75% and 25%; 50% and 50%; 25% and 75%; and 0% and 100%) and is shown in Fig 2A. The fold ratios were 0.83 ± 0.08, 0.49 ± 0.01, 0.67 ± 0.02, 0.70 ± 0.10, and 0.85 ± 0.13, respectively. No significant difference (*p* > 0.05) was observed in all treatment groups compared to those of control groups, although α-TCP and TRF at the ratio of 0:100, exhibited the highest expression of all the treatment groups. This indicated that the presence of H_2_O_2_ in neuronal cells induced the expression of *TTPA* in α-TCP and TRF at the ratio 0:100. However, this was not considered to be statistically significant (*p* > 0.05). Fig 2B shows the expression of *TTPA* in resting neuronal cells supplemented with different percentages of α-TCP and TRF (100% and 0%; 75% and 25%; 50% and 50%; 25% and 75%; and 0% and 100%), where the obtained expression levels were 0.73 ± 0.13, 0.57 ± 0.11, 0.48 ± 0.04, 1.06 ± 0.19, and 2.13 ± 0.58, respectively.

As the concentration of α-TCP decreased, the expression of *TTPA* also decreased. On the other hand, as the concentration of TRF increased, *TTPA* expression also increased. Cells supplemented with 100% TRF showed the highest expression. A significant difference (*p* < 0.05) was recorded when 100% TRF was compared with 100%, 75%, and 50% α-TCP. Results revealed that the oxidative stress increased the expression of the *TTPA* gene when 25% and 0% α-TCP were available to the neuronal cells. This indicated that the gene expression of *TTPA* was increased especially when treated with 100% TRF in order to scavenge α-TCP from the media.

Several studies have shown that oxidative stress may induce the expression of α-TTP. α-TTP expression was up-regulated during oxidative stress in human choriocarcinoma cells [22] and in a rat model with type II diabetes [19]. However, the results were not substantiated while the effects of oxidative stress on the expression of α-TTP in liver [24, 37] were examined. Reasons for these incompatible findings are yet to be established. However, Miyazaki et al. [19] hypothesized that oxidative stress is induced by different pathological conditions and thus leads to variations in the hepatic α-TTP expressions. The mechanisms that regulate α-TTP expression by oxidative stress in the liver requires further investigation.

### Supernatant protein factor gene (SEC14L2) expression in resting and H_2_O_2_-induced neuronal cells

**Fig 3:**
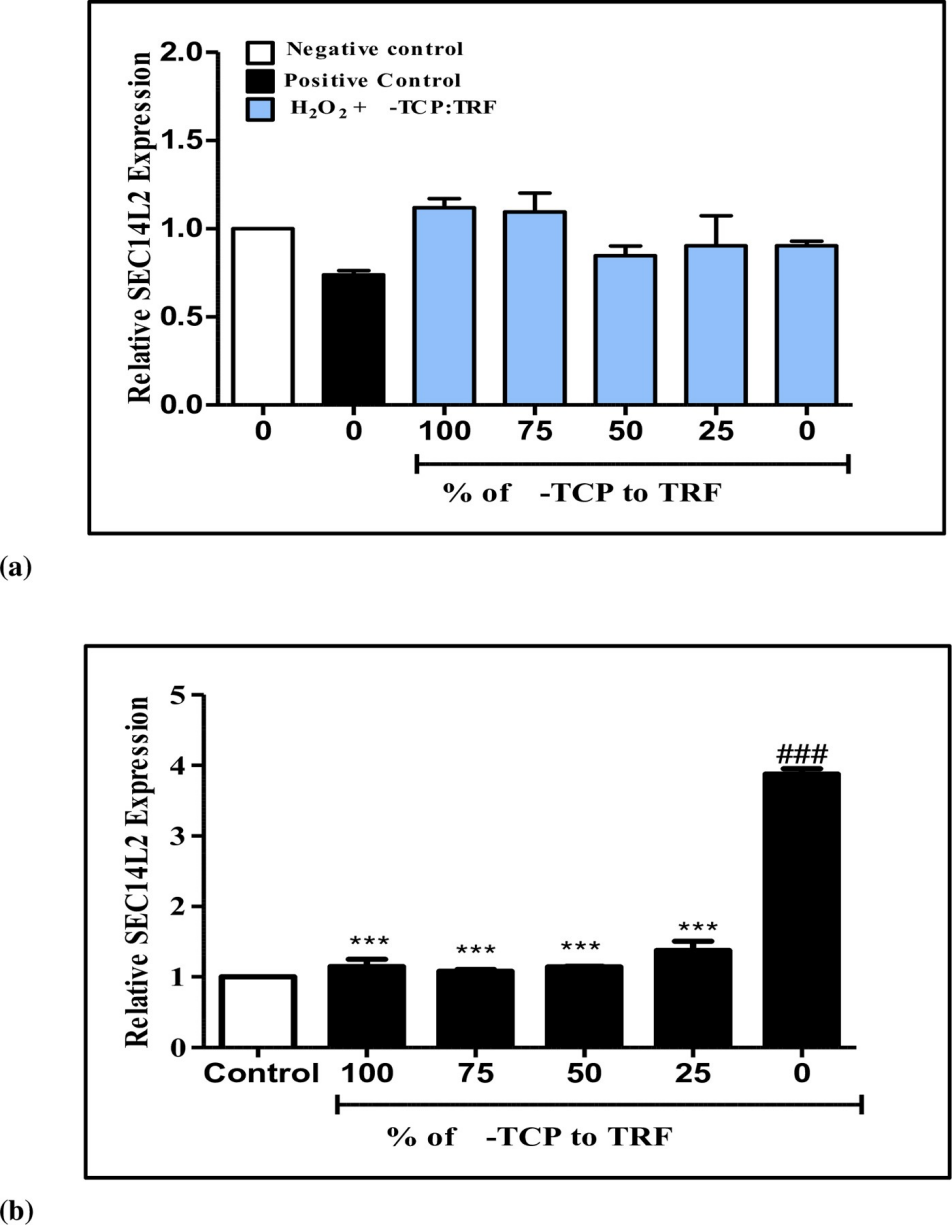
Quantification of *SEC14L2* gene expression in (**a**) neuronal cells treated with 50 μM H_2_O_2_ and (**b**) neuronal cells rested for an hour followed by supplementation with different percentages 200 ng/mL α-TCP and TRF for 4 hours, after which RNA was isolated. Negative control is untreated cells while positive control is cells treated with H_2_O_2_. ^###^*p* < 0.001 vs. control; ***p < 0.001 vs. 0% α-TCP; determined by one-way ANOVA with a Tukey’s post hoc test. Fold change of *SEC14L2* was normalized to *GAPDH*. Results are expressed as mean ± SEM of three independent experiments.

The expression levels of *SEC14L2* in neuronal cells (Fig 3A) exposed to oxidative stress supplemented with different percentages of α-TCP and TRF (i.e., 100% and 0%; 75% and 25%; 50% and 50%; 25% and 75%; and 0% and 100%) had fold ratios of 1.15 ± 0.10, 1.08 ± 0.02, 1.14 ± 0.01, 1.38 ± 0.13, and 3.88 ± 0.08, respectively. Increased fold ratios of *SEC14L2* were observed at high concentrations of TRF. Nonetheless, no significant differences (*p* > 0.05) were found in all treatment groups at different percentages when compared to those of control groups, although 100% α-TCP and 100% TRF exhibited the highest expressions. It is highly likely that the relative expression of the *SEC14L2* gene increased after being treated with α-TCP and TRF at all different doses. Fig 3B showed the expression of *SEC14L2* in resting neuronal cells supplemented with different percentages of α-TCP to TRF (i.e., 100% and 0%; 75% and 25%; 50% and 50%; 25% and 75% and 0% and 100%) obtained respective fold ratios of 1.18 ± 0.07, 1.09 ± 0.02, 1.15 ± 0.01, 1.42 ± 0.09, and 3.90 ± 0.05. The highest significant difference was observed when 100% TRF was compared with the corresponding control and all treatment groups. The highest expression was obtained when 100% TRF was present in the cells.

It was suggested that oxidative stress stimulates the expression levels of *TTPA* and *SEC14L2* genes and this may have been due to the diminishing level of α-TCP since radical scavenging activity have been taking place in the cell, with *TTPA* increasing its expression as the demand for α-TCP increases [37]. Our results showed that in neuronal cells, the expression of the *TTPA* gene was sensitive to oxidative stress. This suggests that the regulation of *TTPA* in neuronal cells affects the distribution of vitamin E in the nervous system. Furthermore, it is reasonable to hypothesize that under conditions of oxidative stress, increased *TTPA* levels may increase α-TCP secretion from the neuronal cells, and proteins have been modified, considering that in the absence of α-TCP, they may have changed in order to taking up TCT.

### Expression of phosphatidylinositol transfer protein α gene (PI-TPNA) in resting and H_2_O_2_-induced neuronal cells

**Fig 4:**
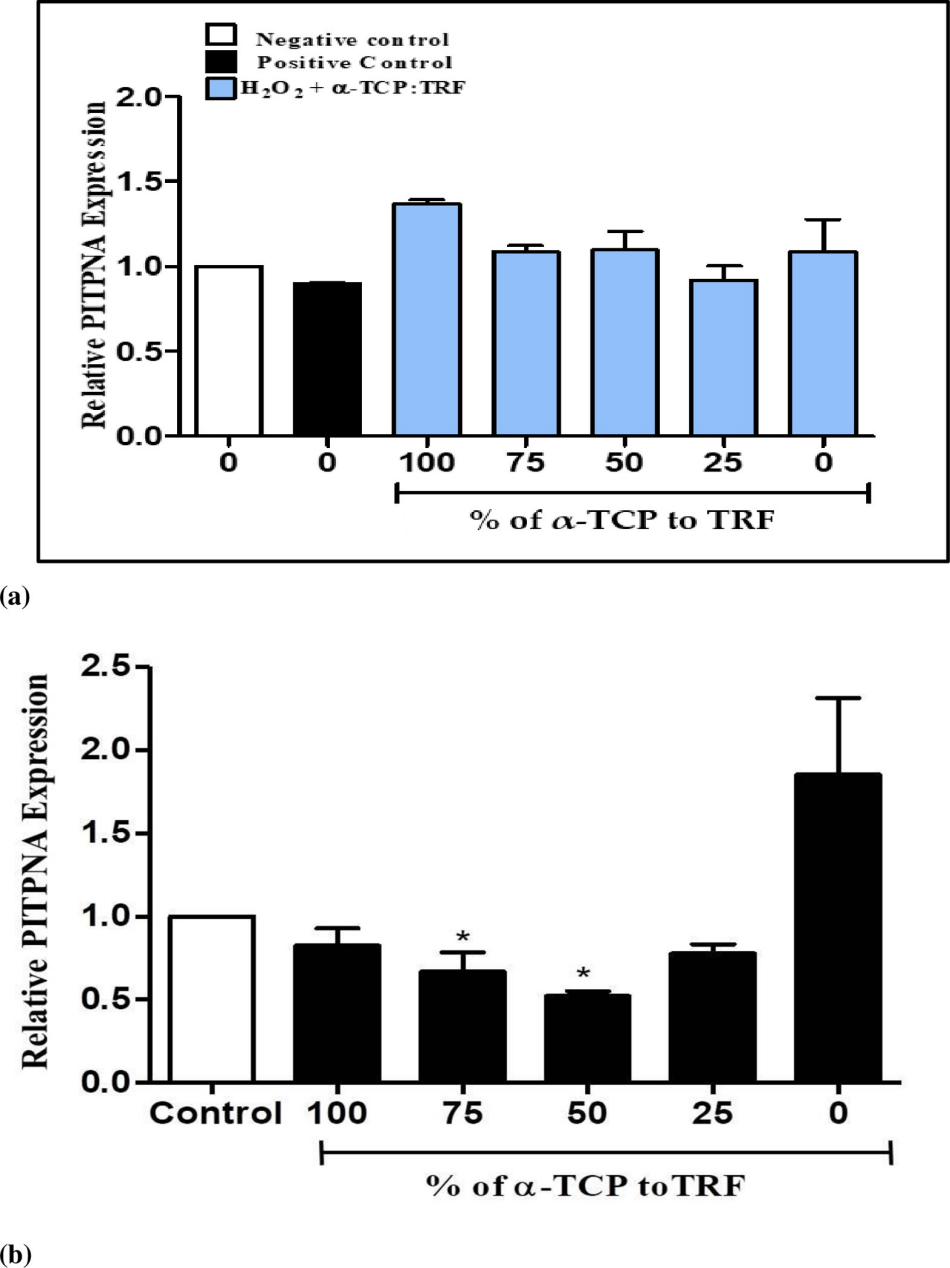
Quantification of *PI-TPNA* gene expression in (**a**) neuronal cells treated with 50 μM H_2_O_2_ and (**b**) neuronal cells rested for an hour followed by supplementation with different percentages of 200 ng/mL α-TCP and TRF for 4 hours, after which RNA was isolated. Negative control is untreated cells while positive control is cells treated with H_2_O_2_. Fold change of *PI-TPNA* was normalized to *GAPDH*. Results are expressed as mean ± SEM of three independent experiments. *p < 0.05 vs. positive control; determined by one-way ANOVA with a post hoc Tukey’s test.

The relative *PI-TPNA* expression in neuronal cells induced with oxidative stress supplemented with different percentages of α-TCP and TRF (i.e., 100% and 0%; 75% and 25%; 50% and 50%; 25% and 75%; and 0% and 100%) is shown in Fig 4A. The fold ratios obtained were 1.36 ± 0.03, 1.15 ± 0.049, 1.20 ± 0.062, 0.93 ± 0.05, and 1.23 ± 0.04, respectively. *PI-TPNA* expression levels were not found to be significantly different (*p* > 0.05) from the control group. The group supplemented with 100% α-TCP exhibited the highest expression levels when compared to positive and negative controls, but the increase was not significant (*p* > 0.05). The relative *PI-TPNA* expression in resting neuronal cells supplemented with different ratios of α-TCP and TRF (i.e., 100% and 0%; 75% and 25%; 50% and 50%; 25% and 75%; and 0% and 100%) is depicted in Fig 4B. The fold ratios were 1.37 ± 0.02, 1.09 ± 0.04, 1.09 ± 0.11, 0.92 ± 0.08, and 1.09 ± 0.20, respectively. A significant decrease (*p* < 0.05) in 75% and 25% and 50% and 50% groups were observed when compared to the 100% TRF group. Further decrease of α-TCP resulted in a slight increase in *PI-TPNA* expression, with the highest value observed for 100% TRF. However, the increase was not significant (*p* > 0.05).

Resting neuronal cells that were supplemented with 100% α-TCP showed no alterations in the levels of *TTPA*, *SEC14L2*, and *PI-TPNA*, indicating that a considerable quantity of α-TCP have been retained within the cells, and that only a small amount of α-TCP was probably used by the cells. This may have been due to the fact that fat-soluble vitamins are retained in the body for a longer period of time than water-soluble vitamins [12]. Furthermore, plasma levels of vitamin E did not respond linearly to changes in intake. For example, when vitamin E supplementation was increased from 400 to 800 mg/day, the resulting increase in plasma TCP was only minor (<10%), suggesting that there are other factor(s), besides intake, which regulate and limit vitamin E levels, and that these factor(s) become saturated at certain intake levels [12].

Studies have shown that affinity for α-TTP to bind α-TCP is 100% as compared to those of 13% of TCT [36]. Increased expression of *TTPA* and *SEC14L2* in the 100% TRF group when the cells were at rest, suggests that in the deficiency of α-TTP-preferred ligand (i.e., α-TCP), the cells increase α-TTP gene expression in order to increase the distribution of α-TCP to cells. This is in agreement with the results of Ulatowski et al. [23], which reported that oxidative stress increased *TTPA* expression in astrocyte cells that referred to TCP, which is distributed by the *TTPA*-expressing astrocytes in order to protect neurons from oxidative stress-induced damages.

### Quantification of vitamin E uptake by neuronal cells using HPLC

**Fig 5:**
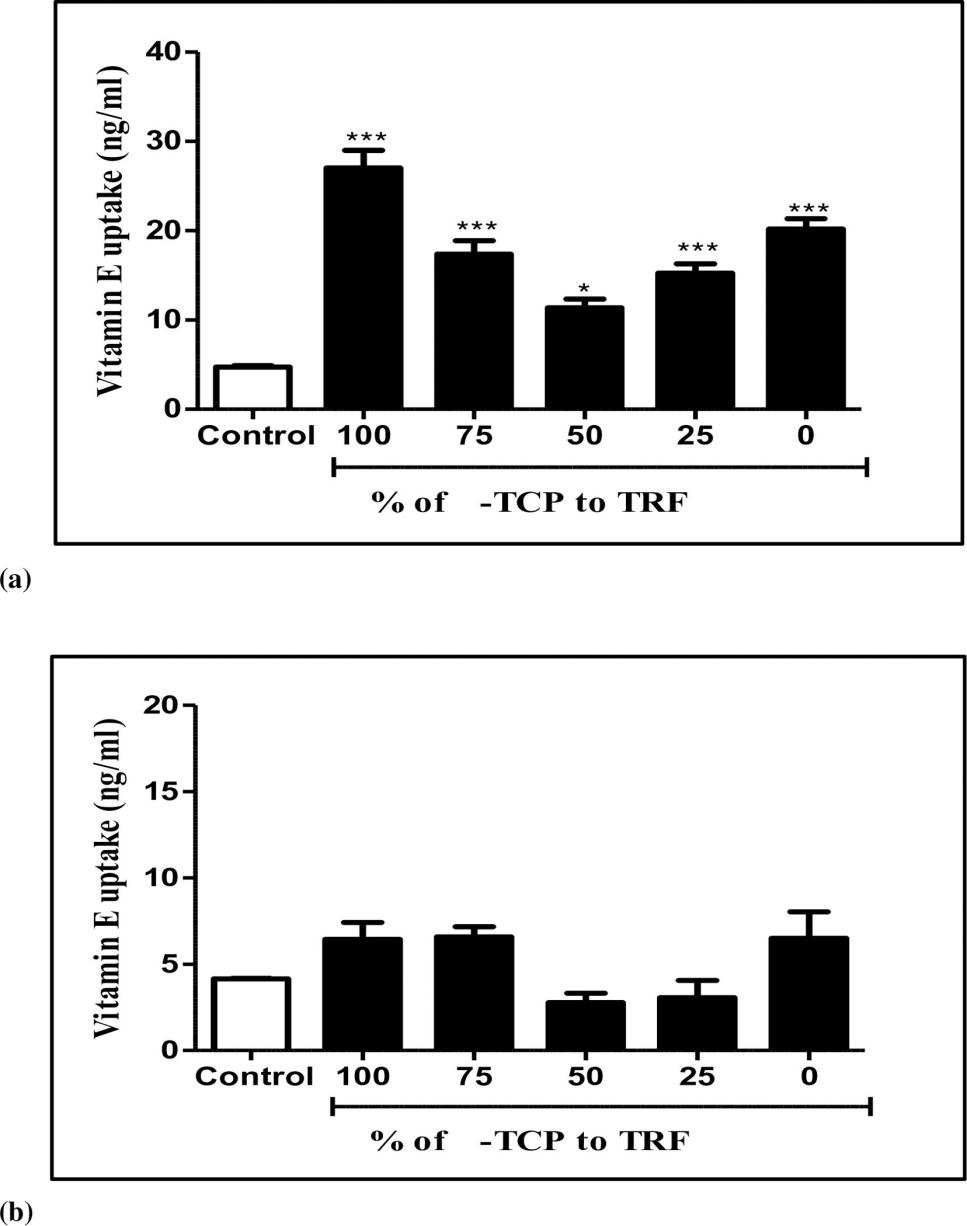
Graphical representation of the amounts of vitamin E uptake by (**a**) neuronal cells induced with 50 μM H_2_O_2_ and (**b**) resting neuronal cells supplemented with different percentages of 200 ng/mL α-TCP and TRF for 4 hours, after which vitamin E was extracted. Results are expressed as mean ± SEM of three independent experiments. ***p < 0.001, **p < 0.01 vs. control; **p < 0.01, * *p* <0.05 vs. 100% α-TCP; determined by one-way ANOVA with post hoc Tukey’s test.

**Fig 6:**
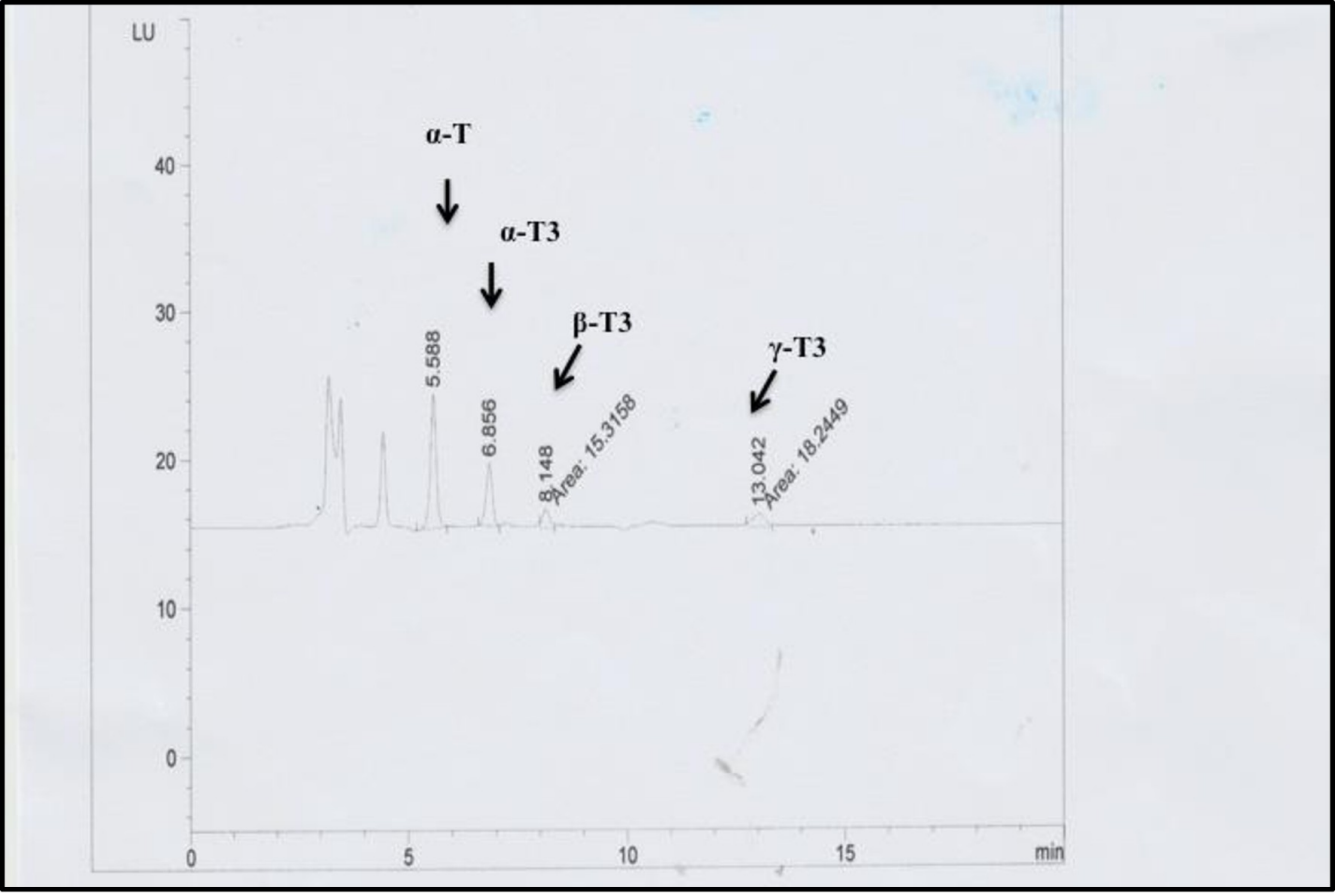
HPLC-fluorescence chromatograms of *R*-α-tocopherol and different forms of tocotrienols released from neuronal cells. Cells were grown for 4 h in the presence of different percentages of 200 ng/mL α-TCP and TRF. Thereafter, medium was collected, and the isoforms were extracted. Peaks α-T, α-T3, β-T3, γ-T3, and δ-T3 correspond to α-tocopherol, α-tocotrienol, β-tocotrienol, γ-tocotrienol and δ-tocotrienol, respectively, as confirmed by authentic standards.

To test if the increased expression of genes in the cells was due to the greater bioavailability of vitamin E; the amount of vitamin E uptake by neuronal cells was determined. Fig 5A showed the uptake of vitamin E in neuronal cells that were exposed to 50 μM H_2_O_2_ for one hour followed by supplementation with different percentages of 200 ng/mL α-TCP and TRF for four hours. Vitamin E uptake by the cells, when they were treated with 100% α-TCP, 75% α-TCP, and 100% TRF increased by 6-fold, 4-fold, and 5-fold, respectively, than the corresponding control (Fig 5A). These data indicated that higher cellular retention of vitamin E was associated with increased *PI-TPNA* and *SEC14L2* gene expression in neuronal cells. Next, cellular vitamin E content was assessed in resting neuronal cells exposed to different percentages of 200 ng/ml α-TCP and TRF for four hours. As shown in Fig 5B, the cellular vitamin E uptake rates were 6.44, 6.59, 2.77, 3.06, and 6.5 ng/mL when neuronal cells were treated with 100% α-TCP, 75% α-TCP, 50% α-TCP, 25% α-TCP, and 0% α-TCP, respectively. The characterization of different forms of vitamin E that were taken up by the neuronal cells were carried out using HPLC, as shown in Fig 6.

### Comparison between *TTPA*, *SEC14L2*, and *PI-TPNA* gene expression and vitamin E uptake in resting and H_2_O_2_-induced neuronal cells

Table 3 showed the comparison between *TTPA*, *SEC14L2*, and *PI-TPNA* gene expression and vitamin E uptake in neuronal cells that were induced by oxidative stress. When neuronal cells were supplemented with 100% α-TCP, the expression levels of *SEC14L2* and *PI-TPNA* increased, and there was a concomitant increase in the ability of cells to retain α-TCP and TRF (Table 3). Furthermore, an increase in the retention of α-TCP and TRF at the cellular level was associated with increased expression levels of the *SEC14L2* gene when neuronal cells were treated with 50% and 75% α-TCP (Table 3). Increased cellular retention of α-TCP and TRF was associated with increases in the relative expression of both *PI-TPNA* and *SEC14L2* genes when neuronal cells were given 0% α-TCP (Table 3). It is highly likely that the decrease in the expression of the *TTPA* gene might have been due to oxidative damages to the neuronal cells that were induced by H_2_O_2_ (Table 3). Table 4 showed a comparison between *TTPA*, *SEC14L2*, and *PI-TPNA* gene expression and vitamin E uptake in resting neuronal cells. When neuronal cells were treated with 100% and 75% α-TCP supplementation, low expression levels of *TTPA*, *SEC14L2*, and *PI-TPNA* was not observed with increased cellular α-TCP and TRF contents (Table 4). Low uptake of α-TCP and TRF was not correlated with low expression levels of *TTPA*, *SEC14L2*, and *PI-TPNA* when neuronal cells were supplemented with 50% α-TCP. High uptake of α-TCP and TRF was correlated with high gene expression levels of *TTPA*, *SEC14L2*, and *PI-TPNA* when neuronal cells were supplemented with 0% α-TCP (Table 4).

**Table 3:** Comparison between *TTPA*, *SEC14L2*, and *PI-TPNA* gene expression and vitamin E uptake in neuronal cells induced with H_2_O_2_.

Percentage of α-TCP to TRF	*TTPA*	*SEC14L2*	*PI-TPNA*	Vitamin E
100%	↓	↑	↑	27.03
75%	↓	↑	↓	17.37
50%	↓	↑	↓	11.36
25%	↓	↑	↓	15.26
0%	↓	↑	↑	20.18

↑ = upregulation; ↓ = downregulation.

**Table 4:** Comparison between *TTPA*, *SEC14L2*, and *PI-TPNA* gene expression and vitamin E uptake in resting neuronal cells.

Percentage of α-TCP to TRF	*TTPA*	*SEC14L2*	*PI-TPNA*	Vitamin E
100%	↓	↓	↓	6.44
75%	↓	↓	↓	6.59
50%	↓	↓	↓	2.77
25%	↓	↑	↓	3.06
0%	↑	↑	↑	6.51

↑ = upregulation; ↓ = downregulation.

By and large, this study found that increased expressions of *TTPA*, *SEC14L2*, and *PI-TPNA* enhanced vitamin E uptake in the neuronal cells. In fact, Vitamin E has eight iso-forms: namely α, β, γ, δ-TCPs and α, β, γ, δ-TCTs. Natural α-TCP is preferentially retained in the human body and this preference is determined by hepatic α-TTP because of its high affinity towards α-TCP. In contrast, SPF *(SEC14L2)* has the same affinity to bind vitamin E and some phospholipids [38, 39]. It has been reported that SPF *(SEC14L2)* has high affinity to bind α-tocopherylquinone—an oxidized product of α-TCP—compared to α-TCP. Taken together, these studies suggest that vitamin E uptake may be modulated by *SEC14L2*, *PI-TPNA*, and *TTPA* in neuronal cells. An increase in gene expression in neuronal cells suggesting that these proteins may facilitate vitamin E transport into neurons and from plasma to retain a high concentration of vitamin E within the cells [11, 30]. It showed that there is an enhanced uptake of vitamin E into the tissues after the consumption of dietary palm oil, especially in the brain. Thus, the bioavailability of vitamin E in the nervous system allows it to carry out its functions [6]. The current study also complements the findings of a study on the cellular uptake of vitamin E in cultured liver cells, corroborating that the structures of neither vitamin E nor α-TTP play a role in mediating the distribution of vitamin E either in the form of tocopherol or tocotrienol [40]. It may be mentioned here that other genes related to vitamin E binding (i.e., *SEC14L2*, *PI-TPNA*, and *TTPA)* are involved in active vitamin E trafficking rather than being only based on passive diffusion, as suggested by Irías-Mata et al. [26].

Further studies are needed to investigate the structural and conformational changes of α-TTP upon binding to different isomers of TCT. Future studies should also be carried out to determine the uptake of vitamin E isomers after having used some pharmacological inhibitors of the ABCA1 transporters, apoA1 and SR-B1, since ABCA1 and SR-B1 are essential components involved in α-TTP-mediated vitamin E uptake when vitamin E is complexed with lipoproteins [14]. The expression levels of the ABCA1, apoA1, and SR-B1 genes should also be examined in order to gain insight into the molecular mechanism of vitamin E uptake by proteins in order to verify the findings of this study.

## Conclusions

This study showed that the expression levels of vitamin E-binding proteins, including α-tocopherol transfer protein (α-TCP/*TTPA*), supernatant protein factor (SPF/*SEC14L2*), and phosphatidyl-inositol transfer protein (PI-TP/*PI-TPNA*), in 0% α-TCP positively correlated to the cellular levels of vitamin E in resting neuronal cells. Generally, current results suggest that the expression levels of vitamin E transport proteins likely influence the cellular vitamin E concentration in neuronal cells. The above results suggest that the regulation of *TTPA*, *SEC14L2*, and *PI-TPNA* genes in neuronal cells affect the distribution of vitamin E in the nervous system and, the whole body as well.

## Abbreviations

α-TCP: alpha tocopherol
α-TCT: alpha tocotrienol
α-TTP: alpha tocopherol transfer protein
ATCC: American Type Culture Collection
ABCA1: ATP-binding cassette transporter subtype A1
AVED: ataxia with vitamin E deficiency
DMSO: Dimethyl sulfoxide
ELISA: enzyme-linked immunosorbent assay
HDL: high-density lipoprotein
H_2_O_2_: Hydrogen peroxide
HPLC: high performance liquid chromatography
LDL: low-density lipoprotein
MTT: 3-(4,5-dimethylthiazolyl-2)2,5-diphenyltetrazolium bromide
PI-TPNA: Phosphatidyl inositol transfer protein alpha gene
PI-TP: Phosphatidyl inositol transfer protein
PBS: phosphate-buffered saline
TAP: tocopherol-associated proteins
TBP: tocopherol-binding proteins
TRF: tocotrienol-rich fraction
VLDL: very-low-density lipoprotein
